# Breaking down Complex Saproxylic Communities: Understanding Sub-Networks Structure and Implications to Network Robustness

**DOI:** 10.1371/journal.pone.0045062

**Published:** 2012-09-28

**Authors:** Javier Quinto, Ma. Ángeles Marcos-García, Cecilia Díaz-Castelazo, Víctor Rico-Gray, Hervé Brustel, Eduardo Galante, Estefanía Micó

**Affiliations:** 1 Centro Iberoamericano de la Biodiversidad CIBIO, Universidad de Alicante, Alicante, España; 2 Instituto de Ecología, A.C. Xalapa, Veracruz, México; 3 Université de Toulouse, École d’Ingénieurs de Purpan, INPT, Toulouse, France; University of Arizona, United States of America

## Abstract

Saproxylic insect communities inhabiting tree hollow microhabitats correspond with large food webs which simultaneously are constituted by multiple types of plant-animal and animal-animal interactions, according to the use of trophic resources (wood- and insect-dependent sub-networks), or to trophic habits or interaction types (xylophagous, saprophagous, xylomycetophagous, predators and commensals). We quantitatively assessed which properties of specialised networks were present in a complex networks involving different interacting types such as saproxylic community, and how they can be organised in trophic food webs. The architecture, interacting patterns and food web composition were evaluated along sub-networks, analysing their implications to network robustness from random and directed extinction simulations. A structure of large and cohesive modules with weakly connected nodes was observed throughout saproxylic sub-networks, composing the main food webs constituting this community. Insect-dependent sub-networks were more modular than wood-dependent sub-networks. Wood-dependent sub-networks presented higher species degree, connectance, links, linkage density, interaction strength, and were less specialised and more aggregated than insect-dependent sub-networks. These attributes defined high network robustness in wood-dependent sub-networks. Finally, our results emphasise the relevance of modularity, differences among interacting types and interrelations among them in modelling the structure of saproxylic communities and in determining their stability.

## Introduction

Network analysis is a valuable tool for studying the diversity of species and interactions in large trophic networks [1]. A high number of ecological communities have been studied under this perspective, discovering specialised interacting patterns as nestedness in mutualistic networks [2], [3] or modularity in antagonistic networks [4], providing insight into the function and evolution of the components of the system [5]. Specialized interacting patterns act like a variable modelling the network structure of interactions, reducing the effective interspecific competition and enhancing the number of coexisting species [6]. The biotic environment of co-occurring species critically determines the way in which species adapt to new environments [7], as antagonistic and facilitative interactions between species determining the response to environmental perturbations [8].

Research on ecological communities has been dominated by small-scale studies [9], and restricted to a single type of interaction [4], while only recently, spatio-temporal scales of ecological communities [10]–[12] or complex networks with different types of interaction [13], [14] have been addressed with network analysis. Ecological network studies are largely focussed on qualitative data, assuming that all interacting species are equally important [15]. However, specialised network patterns are best defined at quantitative scale in both mutualistic and antagonistic communities [16], and the relative abundances of the components of the networks influences structural patterns as asymmetry [17].

One of the most complex communities in terrestrial environments develops inside tree hollows, which provide a diverse range of microhabitats within forest ecosystems [18]–[21]. In each tree hollow, saproxylic insect assemblages with a high number of species with several types of interaction coexist: dependence or not of woody resources or/and feeding guilds. Moreover, from a functional perspective, saproxylic insects include a large number of taxa that play a key role in the decomposition of woody material in forest ecosystems [22]. Nevertheless, the diversity of their interactions is poorly understood [23] and consequently saproxylic insect communities [24] have to be studied form the point of view of interacting networks.

Here we provide a first approach to characterise and to analyse specialized interacting patterns occurring in quantitative tree hollow-saproxylic insect food webs, using network analyses. We used empirical data related to trophic structure of the complex community to break down quantitative saproxylic food webs inhabiting hollow microhabitats of Mediterranean forests in Cabañeros National Park (Spain). Mediterranean forests have a large number of woody species compared to central or northern Europe [25] and host a high animal diversity [26], where saproxylic insects make up the highest percentage of their biodiversity. In order to incorporate the high amount of the components of the tree hollow/saproxylic insect interaction, we have included the most representative tree species of the woodland in the studied area. Among the Coleoptera and Diptera (Syrphidae) saproxylic species coexisting in tree hollows, we considered three levels of interaction: 1) complete network (the ‘whole’ saproxylic community), 2) sub-networks defined according to the use or not of woody resources (direct or indirect saproxylics), and 3) sub-networks according to their feeding guild (xylophagous, saprophagous, xylomycetophagous, predators and commensals). In particular we addressed the following questions: i) How are saproxylic sub-networks organized either defined by the use of resource and by the feeding guild or according to specialised patterns of interaction, as nestedness or modularity? ii) Are there differences in interacting and ecological patterns among sub-networks; and iii) Which are the implications of these properties in network robustness, from random and directed simulations of the lost of tree hollow microhabitats?

## Methods

### Study Site and Sampling

The study was conducted in Cabañeros National Park (39° 23′ 47″ N; 4° 29′ 14″ W; altitude varies between 560 and 1448 m), a natural area of 40856 ha located in central Spain. The climate is Mediterranean, the annual average temperature fluctuates from 12.9 to 15.6°C and the annual precipitation averages between 500 and 750 mm [27].

The park is constituted by extensive areas of well-preserved Mediterranean landscape, with various woodland types [27].Field work was carried out in the most representative Mediterranean forests of the National Park: sclerophyllous forest of holm-oak *Quercus rotundifolia* Lam., mixed deciduous forest dominated by Pyrenean oak *Quercus pyrenaica* Willd. and the native oak *Quercus faginea* Lam., and riparian forest of narrow-leafed ash *Fraxinus angustifolia* Vahl. To capture saproxylic insects breeding and inhabiting tree hollows we used emergence traps specially modified from Colas [28]. Every tree hollow was covered with acrylic mesh and sealed up with staples. Specimens emerged and come into a white collecting pot containing ethylene glycol as preservative [21], [28]. In every forest type we selected 30, 30 and 27 hollow trees, respectively. The first indispensable necessity for study basic specialized patterns occurring on saproxylic communities inhabiting this ecological niche was to represent the real heterogeneity and abundance of tree hollows in each woodland type, always having account the high degree of protection of this National Park and the inherent need to protect and conserve this important and limited microhabitat. We considered a maximum of 30 tree hollows representing the natural proportion per woodland type, including multiple ecological variables able to model saproxylic communities at microhabitat scale in the studied area, as hollow size, hollow position, tree diameter, etc. [30], [18], [19]. This passive method of capture allows recording saproxylic species shortly after their emergence from immature stages, offering a representative outline of the linkage of any recorded species to this microhabitat, being the interaction strength a good surrogate of this linkage. Collecting tubes were replaced every month throughout a year (February 2009–March 2010).

### Identification of Selected Taxa

We selected Coleoptera and Diptera as study groups at the hollow level, because they are the best known and represented groups in forests [30]–[32], allowing us to study the network properties from a quantitative point of view. We considered the Syrphidae as a bioindicator of species and interaction richness among the Diptera, because i) they have been traditionally used next to beetles in studies concerning saproxylic insects [33], [34], and present a high number of saproxylic species around Europe [33], using a wide range of microhabitats [35], what has led them to be used as indicators of woodland quality [24], [34], to be flagships for the conservation of the wider community of saproxylic organisms [35] or to be included in national red lists [36], and ii) they represent the best studied family (or just the unique) of Diptera in the study site, presenting high number and abundance of mainly exclusive saproxylic species highly strengthened with tree hollow microhabitats [20].

Identification of Coleoptera families was done using Delvare and Aberlenc keys [37], and for species identification of many families we also counted with the help of invited specialists (see Acknowledgments). Syrphids were identified using the van Veen [38] and Speight keys [39].

### Classification into Levels of Interaction

Saproxylic communities are complex networks involving different types of interactions that depend on different trophic resources available inside tree hollow microhabitats. Because of the large number of both tree hollows and species nodes, we began breaking down the crude network into smaller sub-networks, recording biological/ecological information available for this saproxylic functional group, using the bibliography, the ‘Frisbee’ data base [40] and expert’s information (see Acknowledgements). Clear facultative associations and species with unknown biology were removed for the analyses. For this objective, we classified the saproxylic entomofauna according to the main ecological guilds described by Speight [24] and Bouget et al. [41]: xylophagous, xylomycetophagous, saprophagous, predators and commensals (Table S1). Finally, based on the use of trophic resources on hollow trees, we classified the whole saproxylic community in two basic levels: 1) according to the type of interaction, a) direct saproxylic insects (wood-dependent), feeding on woody resources, as dead or dying wood, sap run or wood-inhabiting fungi, and b) insect-dependent sub-networks (insect-dependent), inhabiting tree hollows but mainly depending on the activity or presence of other saproxylic insects for their development: predators and commensals., and 2) according to trophic guilds: i) xylophagous, ii) saprophagous, iii) xylomycetophagous (wood-dependent sub-networks), and iv) predators and v) commensals (insect-dependent sub-networks).

### Network Analysis and Statistics

#### Modularity

We used Aninhado [3] to analyse the existence of nestedness patterns (nestedness as NODF estimator), generating 1000 replicates for each saproxylic sub-network with a CE null model. CE considers that the probability of an interaction is proportional to the generalisation level of both species, so allowing evaluating the influence of abundances to nestedness pattern.

To study modularity we used ‘netcarto’ [5] and Pajek [42]. For a given partition of the nodes of a network into modules, the modularity *M* of this partition is [43]–[45]:
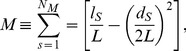
(1)where *N_M_* is the number of modules, *L* is the number of links in the network, *l_s_* is the number of links between nodes in module *s*, and *d_s_* is the sum of the degrees of the nodes in module *s*. This heuristic module identification algorithm finds the minimum partition into modules. A good partition of a network must comprise many within-module links and as few as possible between-module links. Equation (1) does that by imposing that *M* = 0 if nodes are placed at random into modules or if all nodes are in the same cluster [5], [43]–[45]. We assessed the simulated annealing procedure to find the optimal partition with largest modularity of the network into modules [46]. This stochastic optimization technique enables to find ‘low-cost’ configuration without getting trapped in ‘high-cost’ local minima, by means of the introduction of computational temperature *T*. When *T* is high, the system can explore configurations of high cost whereas at low *T* the system only explores low-cost regions. By starting at high *T* and slowly decreasing *T*, the system descends gradually towards deep minima, eventually overcoming small cost barriers. When identifying modules, the objective is to maximize the modularity, and thus the cost is *C = –M*, where *M* is the modularity as defined in equation (1). At each temperature, we perform a number of random updates (1000, *f* (iteration factor) = 0.1, *c* (cooling factor) = 0.995) and accept them with probability *p* [47]:
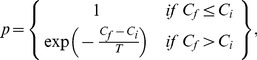
(2)where Cf is the cost after the update and Ci is the cost before the update.

We also used ‘netcarto’ to heuristically describe the differences on the composition and interrelations among modules along 25 randomisations maximizing modularity (subjective benchmark). We considered that the partition with a lesser number of modules could be used as the ‘minimum partition with largest optimisation of modularity’ for each randomised sub-network, from we can know the main minimal subsets of interacting nodes or sub-modules of any modular network. Based on the connectivity in these resultant sub-modules, we yielded and studied the cartographic representation of the complex network [43]. We obtained the within-module degree for each node. If *k_i_* is the number of links of node *i* to other nodes in its module *s_i_*, 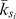 is the average of *k* over all the nodes in *s_i_*, and 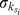
is the standard deviation of *k* in *s_i_*, then:
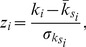
(3)is so-called *Z-score* (z ≥2.5 determines hub nodes, and z <2.5 non-hubs nodes), which measures how well-connected node *i* is to other nodes in the module. To assess the connection of a node to modules other than its own, we obtained the *P-score* or participation coefficient *P_i_* of each node *i* as:



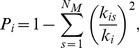
(4)where 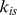 is the number of links of node *i* to nodes in module *s*, and *k*_i_ is the total degree of node *i*. The *P-score* of a node ranges between 0 if all the links are done within-module and 1 if the links are uniformly distributed along the set of sub-modules. According to these values, we then classified each node into *system independent* ‘universal roles’: *kinless hub* (R7), *connector hub* (R6), *provincial hub* (R5), *non-hub kinless* (R4), *non-hub connector* (R3), *peripheral* (R2) and *ultra-peripheral* (R1), analysing their number and distribution along sub-modules and implications on modularity patterns.

#### Interacting patterns

We used ‘R-bipartite’ [48] to quantitatively assess interacting and distributional patterns between trophic levels of each sub-network and the set of tree hollows assessed. Network attributes analysed were links (mean number of links per species [defined as the sum of links divided by the number of species]), species degree (the sum of the diversity of links per species), interaction strength (sum of dependencies for each species), connectance (the proportion of realised links of the total possible in each network [defined as the sum of links divided by the number of cells in the matrix]), linkage density (a quantitative measure defined as the mean number of interactions per species), H2′ (a measure of network specialisation [which ranges between 0: no specialisation, and 1: complete specialisation]), V-ratio (Variance-ratio of species numbers to individual numbers within species for the higher trophic level [values larger than 1 indicate positive aggregation or association, values between 0 and 1 indicate disaggregation of species]).

#### Robustness to microhabitat extinction

We assessed microhabitat relevance throughout the simulation of primary extinction (slope-estimation derived from randomly removing tree hollow nodes of the lower trophic level) and secondary extinctions approach [49] [slope of the secondary extinction sequence to species in the higher trophic level, following an extermination of highly interconnected tree hollows in the lower trophic level]). We only performed network robustness to species extinctions for insects (higher trophic level) because the set of tree hollows (lower trophic level) is not really affected by removing saproxylic insects. We also studied sub-network robustness [50] as a measure of the system to the random and directed lost of tree hollows (the area below a extinction curve, where R = 1 correspond to a curve that decreases very mildly up to the point at which almost all animal species are eliminated, whereas with R = 0 the curve decreases abruptly as soon as any species is lost). The analyses were carried out separately for each sub-network.

## Results

### Characterisation of Saproxylic Sub-networks

We recorded 3680 individuals of Coleoptera belonging to 135 species and 41 families, and 462 individuals of Syrphidae: Diptera belonging to 22 species (Table S1). The complete network was constituted by 244 nodes, corresponding with 157 insect species nodes and 87 tree hollow nodes. The number of saproxylic insect and tree hollow nodes for the rest of the saproxylic sub-networks is reflected in Table 1.

**Table 1:** Ecological and network attributes modelling saproxylic sub-networks.

	Network metrics												
Network	SP	TH	NODF	M	L/S	C	LD	H2	V-ratio	PE	RPE	SE	RSE
Red	158	87	13.11	1	4.82	0.086	*	*	14.72	*	*	*	*
Direct	104	86	15.37	1	4.69	0.099	*	*	16.38	*	*	*	*
Indirect	54	73	11.76	1	2.284	0.074	6.618	0.545	8.97	2.723	0.722	7.48	0.866
Xylophagous	21	80	24.24	1	2.614	0.157	10.321	0.453	20.02	2.1	0.667	6.658	0.857
Saprophagous	45	81	13.93	1	2.575	0.089	7.562	0.542	10.87	2.725	0.72	8.431	0.881
Xylomycetophagous	38	82	17.57	1	2.525	0.097	11.296	0.364	19.27	2.569	0.712	7.878	0.87
Predators	26	66	12.38	2	1.576	0.086	5.568	0.601	6.65	1.499	0.592	4.6	0.793
Commensals	28	61	14.23	3	1.629	0.085	6.241	0.601	11.53	1.74	0.628	5.124	0.825

* Values impossible to obtain because the matrix size blocks the running of the programme.

SP: number of interacting insect species nodes (higher trophic level); TH: number of interacting tree hollow nodes (lower trophic level); NODF: nestedness as NODF estimator; M: number of isolated modules; L/S: links per species; C: connectance; LD: linkage density; H2′: specialisation; V-ratio: variance ratio; PE: extinction slope of higher trophic level for a random extinction (100 replicates); RPE: robustness for a random extinction; SE: secondary extinction slope of the higher trophic level for a selective extinction of the most interconnected nodes (100 replicates); RSE: robustness for a directed extinction.

### Modularity and Sub-modularity

The results showed a lack of nested patterns in the studied sub-networks, implying low nestedness values (less than 25% in all cases, P>0.05) (Table 1). Quite the opposite, in all the levels of interaction evaluated we found a modular structure of interactions, which were characterised by the presence of a unique module in the largest sub-networks evaluated at global scale: 1) complete network, 2) direct saproxylic network, indirect saproxylic network, and 3) xylophagous, saprophagous, xylomycetophagous sub-networks. Furthermore, in predator and commensal sub-networks we found two and three modules, respectively, which were characterised by a main module housing the majority of interactions, and few isolated modules constituted by pairs of interacting species.

For the modularity comparison with randomised networks using simulated annealing procedure, all the sub-networks analysed were statistically significant: complete network (M = 0.255, p = 0.005), direct (M = 0.256, p = 0.006), indirect (M = 0.404, p = 0.009), xylophagous (M = 0.319, p = 0.009), saprophagous (M = 0.371, p = 0.009), xylomycetophagous (M = 0.35, p = 0.009), predator (M = 0.497, p = 0.012), and commensal network (M = 0.471, p = 0.012). The analyses of these sub-networks revealed the existence of a variable number of sub-modules in all the assessed sub-networks (Table 2). The complete network was composed of five to eight sub-modules, but seven sub-modules was the most supported result (48%). Direct sub-network was composed of six to eight sub-modules, but six and seven modules were the most supported (44% and 48%, respectively). Indirect sub-network was composed of six to eight sub-modules (seven and eight modules were the more supported 40% and 48% respectively). The xylophagous sub-network was composed of five to seven, being six sub-modules the most supported result (84%); saprophagous sub-network by five to nine sub-modules, being seven sub-modules the most frequent value (64%); xylomycetophagous sub-network of five to seven sub-modules (six sub-modules showed a support of 68%); predator sub-network of seven to 10 sub-modules (eight and nine sub-modules were the best supported 36% and 40%); commensal sub-network of seven to 10, being nine sub-modules, the most common configuration (50% of the results).

**Table 2:** Variation of number of sub-modules.

	Number of sub-modules					
Network	5	6	7	8	9	10
Complete network	4	28	48	20	–	–
Direct	–	44	48	8	–	–
Indirect	–	12	40	48	–	–
Xylophagous	12	84	4	–	–	–
Saprophagous	4	4	64	24	4	–
Xylomycetophagous	12	68	20	–	–	–
Predators	–	–	4	36	40	20
Commensals	–	–	4	16	52	28

Number of sub-modules present in each sub-network, expressed as the percentage of times with the same number of sub-modules from the 25 randomisations arbitrarily considered.

### Analyses and Characterisation of Sub-modules and Roles

The complete network was composed at least by five main interacting sub-modules, in which tree hollows, wood- and insect-dependent species comprised subsets of closely interacting nodes along randomisations. However, the node composition for each sub-module changed along the 25 randomisations considered for the whole network, being more or less variable depending on the sub-module considered. Sub-modules 2, 3, and 4 were the most cohesive sub-modules, and their constituting nodes appeared together in 76%, 88%, and 68% of the times, respectively; whereas sub-modules 1 and 5 were less cohesive, appearing together in 29.17% and 31.71% of the times, respectively.

Sub-module 2 was defined by the high number of saprophagous species interacting with a close subset of tree hollows of the three studied tree species, where Cetoniidae species were related with Tenebrionidae species and with uncommon Syrphidae species. The xylophagous guild was mainly represented by generalist Cryptophagidae species and the xylomycetophagous guild by Laemophloeidae, Latridiidae and Curculionidae species. Associated fauna was characterised of predator species belonging to Elateridae, Trogossitidae, Melyridae and Rhizophagidae. Sub-module 3 was mainly constituted of xylomycetophagous species interacting with holm-oak and ash tree hollows, where generalist species of Scolytiidae and Biphylidae coexisting with specialist Latridiidae, Endomychidae and Silvanidae species. The saprophagous guild was composed of hoverfly species commonly present in thermophylous forests. Sub-module 4 was characterised by a high number of both saprophagous and xylomycetophagous species interacting in tree hollows in deciduous forests. Saprophagous guild was composed by Cryptophagidae, Curculionidae and Syrphidae Diptera species. The xylomycetophagous guild was represented by Anobiidae, Cryptophagidae, Cerylonidae, Latridiidae, Mycetophagidae, Silvanidae, Tenebrionidae and Zopheridae species. Indirect fauna was characterised by predator species belonging to Elateridae and Cryptophagidae families, and by commensal species belonging to Nitidulidae. See Table S2 for know in detail the node composition of these five main sub-modules.

**Figure 1:**
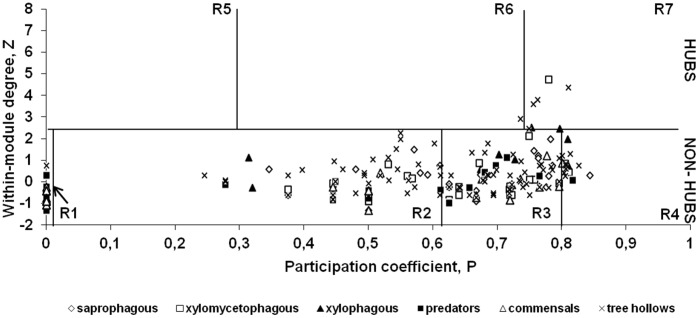
Ecological role diagram. Ecological role diagram for the saproxylic guilds and tree hollows, showing their role distribution along ecological regions in the *z-P* parameter space. This representation showed that the higher proportion of nodes belonged to non-hubs R1 (24.18%), R2 (29.91%), and R3 (37.3%), corresponding with the 91.39% of the nodes, what implicates a constant predominance of weak connections and a similar role composition along sub-modules and saproxylic trophic guilds. The number of hub nodes was low, and they normally corresponded with *kinless hub* (R7) tree hollow nodes heterogeneously connected along sub-modules. So role-to-role connectivity often happened among R1-R2-R3 and in less extent among R7-R1/R2/R3.

Complete network was composed by six ecological roles (Figure 1), corresponding with 6 *hub nodes*: one *connector hub* (R6), and five *kinless hub* (R7); and 238 *non-hub nodes*: 59 *ultra-peripheral* (R1), 72 *peripheral* (R2), 91 *non-hub connector* (R3) and 15 *non-hub kinless* (R4). No *provincial hub* nodes (R5) were present in this ecological network. The distribution of roles was similar among sub-modules. The higher proportion of nodes belonged to non-hubs with ecological roles R1 (24.18%), R2 (29.91%) and R3 (37.3%), comprising the 91.39% of the nodes, and thereby the ‘density landscape’ was displaced towards non-hub region, indicating the high proportion of weakly connected nodes throughout sub-networks. See Table S2 for Z-score, P-score and role for each node.

#### Interacting and distributional patterns

We observed a high variability in the distribution of interactions among the analysed sub-networks. Nodes in the direct sub-network usually presented a higher number of links, connectance, species degree, and interaction strength than indirect sub-networks. Moreover, the three feeding guilds depending on woody resources: xylophagous, saprophagous, xylomycetophagous, also presented higher values in these network metrics than indirect feeding guilds depending on the presence or activity of other saproxylic insects: predators and commensals. These woody-linked trophic levels showed a higher linkage density, because the abundances of both tree hollows and species in these sub-networks were two to three times higher than in saproxylic insect-dependent sub-networks.

All the wood-dependent sub-networks were composed by a higher number of generalist species than saproxylic insect-dependent sub-networks, and these generalist species usually presented higher interaction strength. The xylomycetophagous species *Xyleborus monographus* was the most generalist species, interacting with 61 tree hollows (>70% of possible interactions). The xylophagous guild had the highest number of generalist species, and jointly with saprophagous guild held the highest heterogeneity of associations. The indirect, predator, and commensal trophic levels were composed by a relative lesser number of interactions, and also showed a lower number of generalist species. The commensal guild was more generalist than predators, and presented higher interaction strength in their connexions, as *Prionocyphon serricornis* (Helodidae), *Epuraea fuscicollis* and *Soronia oblonga* (Nitidulidae). As a common pattern, all the trophic levels were constituted by a high proportion of low-linked insects species (1–3 links), ranging from 40 to 48% in woody-linked sub-networks, and from 57 to 68% in saproxylic insect-linked sub-networks. These set of interactions were heterogeneously distributed along the tree hollows.

On the other hand, the most interconnected tree hollow nodes corresponded with large tree hollows, which commonly housed a higher diversity and amount of trophic resources, microhabitats or hosts/preys, and where a diverse ‘team’ of generalist insect species coexist and interacts, being less than the 36% specialist insect species (1 to 3 interactions). The level of specialisation (H2′) differed among guilds (Table 1), being insect-dependent sub-networks (H2′ = 0.545) and overall predator and commensal guilds the most specialised sub-networks (H2′ = 0.601 in both cases). Among wood-dependent sub-networks, xylomycetophagous guild was the less selective in their distributional pattern (H2′ = 0.364). Variance-ratio values were larger than 1 in all cases, indicating positive aggregation of species or competence among species belonging to same trophic guild, being predators the least aggregated guild (V-ratio = 6.65), and xylophagous and xylomycetophagous the most aggregated guilds (V-ratio >19).

#### Robustness to species extinctions

**Figure 2:**
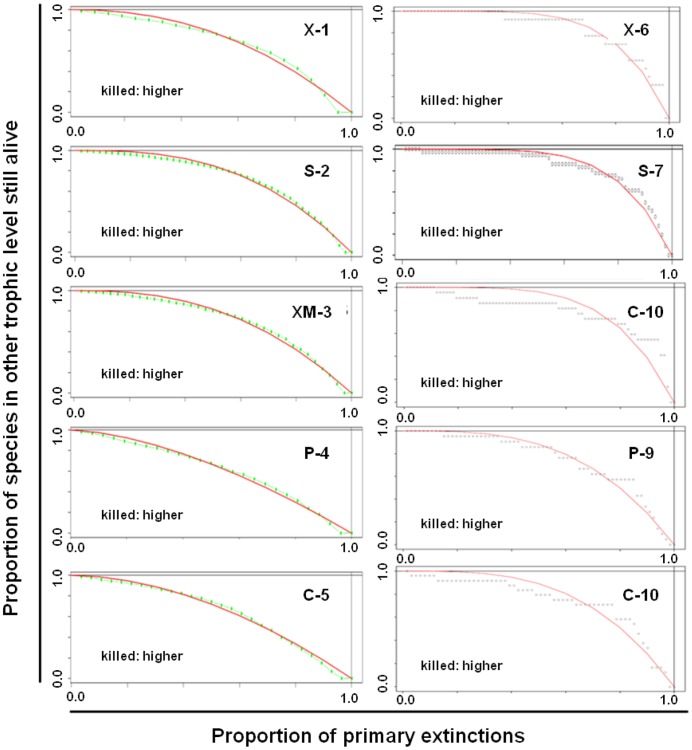
Sub-networks robustness. Random (1–5) and directed (6–10) extinction curve obtained for each feeding guild: xylophagous (X), saprophagous (SX), xylomycetophagous (XM), predators (P), and commensals (C), showing a relative high network robustness to both random and directed extinction simulations in all the trophic levels considered, being wood-dependent guilds more robust than insect-dependent guilds.

Saproxylic sub-networks were moderately robust to tree hollow nodes extermination in both random and directed extinction simulations (see robustness values in Table 1), and most of the insect species survived even if 50% of the tree hollows were eliminated (Figure 2). Both wood- and insect-dependent sub-networks were more vulnerable to a random sequence of losses of tree hollows, presenting lower robustness values than a direct extermination of the most interconnected tree hollows. The wood-dependent sub-network and the feeding guilds constituting them: xylophagous, saprophagous and xylomycetophagous sub-networks, were more robust than insect-dependent sub-networks and their trophic guilds: predators and commensals sub-networks, in both random and directed cascading extinctions.

## Discussion

Results show for first time how a quantitative complex network such as saproxylic community with different types of interaction are organised in interacting food webs, and represent a step forward to understand how sub-networks conforming complex networks may be related. All the saproxylic sub-networks assessed presented a modular pattern of interactions, determining at global scale one large module and a low number of small isolated modules in some of them. Depending on the sub-network, these large modules comprised different numbers of constituent sub-modules, appearing higher number of sub-modules in insect-dependent sub-networks. Wood-dependent sub-networks were more heterogeneously connected: more links, higher species degree, connectance and linkage density, and presented higher interaction strength than insect-dependent sub-networks. Moreover, insect-dependent sub-network and overall their constitutive feeding guilds: predators and commensals, were the most specialised and least aggregated sub-networks. As a consequence, wood-dependent sub-networks (including xylophagous, saprophagous and xylomycetophagous) presented higher network robustness in both random and directed extinction simulations.

### Specialised Interacting Patterns

The majority of the nodes in all the saproxylic sub-networks were within a large and densely interconnected module at global scale, indicating that insect species of a functional module were usually coexisting in a similar subset of tree hollows. Moreover, the number of small modules of a sub-network was related with the number of participating nodes, and insect-dependent sub-networks (with lesser numbers of nodes) presented higher numbers of small isolated modules than wood-dependent sub-networks. This architecture of one large and few isolated modules also occurs in other ecological networks presenting two interaction types [13].

Modularity analysis revealed the existence of closely interacting sub-modules, shaping thereby the main interacting food webs for this saproxylic community. The study of modularity values and number of sub-modules for each sub-network along randomisations, showed a slight variation for modularity values, number of sub-modules and node associations among partitions, indicating the predominance of this specialised pattern along saproxylic sub-networks, and therefore, concluding that the number of randomisations considered using simulating annealing seemed to be a suitable procedure to assess the variation of modularity in complex networks. In the complete network, not all the resulting sub-modules were equally conclusive and solid, but modularity analyses pointed out at least three cohesive sub-modules for this ecological community. This relative high proportion of within-module links also occurs in other modular complex networks [51], showing the number of modules of a networks [5] and determining the ecological niche of their constituent species [11] and therefore preferential interrelations for this saproxylic network, as for instance the high species richness of saprophagous species inhabiting deciduous tree hollows in the sub-module 2, the high species number of saprophagous and xylomycetophagous coexisting and interacting with deciduous tree hollows in sub-module 4, or generalist xylomycetophagous species interacting with saprophagous hoverflies in ash and holm-oak tree hollows in sub-module 3.

The connectivity of the nodes of a complex network enables to classify nodes into universal roles according to their pattern of intra- and inter-module connections [5], [43]. Ecological role distribution was characterised of a variable high proportion of non-hubs nodes, depending on the sub-module size or number of nodes, which entailed a general composition of weak but heterogeneously connected nodes. By this reason, a similar composition of non-hubs nodes occurred among sub-modules, and nodes of the same feeding guild tended to have similar ecological roles [52], therefore presenting similar topological properties [5]. The role composition obtained for this modular network could be associated with the high abundance and heterogeneity of tree hollows characteristic of Mediterranean forests [20], providing a diverse range of microhabitats and availability of trophic resources that allow to establish at least several weak interconnections for each insect species conforming this saproxylic community. In fact, the most interconnected nodes of the whole network mainly corresponded with a limited proportion of big tree hollows (corresponding with the highest internal volumes along the matrix of tree hollows studied, ranging from 0.1 to 0.28 m^3^), which in general housed high amount of trophic resources/microhabitats as dead and decay wood, and therefore hosts/preys for indirect fauna. That big tree hollows are commonly associated with aged trees, which hold the highest numbers of saproxylic species inhabiting Mediterranean forests [53], [22]. The role composition was determinant in supporting associations between pairs of sub-modules along randomisations, implying the relevance of weak connections in maintaining the modular structure and their constant role composition. The heterogeneity inherent to this microhabitat suggests the existence of other fundamental ecological patterns determining the species distribution and modelling interacting patterns, such as the influence of microenvironmental variables associated to tree hollow. Because we have a solid database recording the variation of a large set of ecological variables, our next step would be to conscientiously examine this topic elsewhere.

### Interacting Patterns Conditioning Modularity

The wood-dependent sub-network (including xylophagous, saprophagous and xylomycetophagous) presented higher species degree, connectance, links, linkage density, interaction strength than the insect-dependent sub-network (predators and commensals), comprising a higher heterogeneity of interactions. The resemblances among related sub-networks may be explained not only by the bound of the interaction, but by the similar abundances of weakly connected nodes (corresponding with the high amount of non-hub nodes) among guilds and throughout the matrix of tree hollow and insect species nodes, constituting a high diversity of interactions among nodes and sub-modules. Both wood- and insect-dependent interactions are coexisting in space and time in tree hollows, but they differ in the dependence on microhabitats for their development or establishment of the interaction. These biological and ecological aspects resulted in great differences in the species composition and interacting patterns of each sub-network and sub-module, driving the differences observed in modular patterns (as modularity values or number of sub-modules) of the resulting food webs involving different types of interaction. Antagonistic interactions tend to be organised in modules even when they are densely connected [16]. The xylophagous sub-network presented lower modularity values and number of sub-modules, and were more densely connected among them than the predator sub-network, emphasising clear differences in modularity patterns according to the boundary of the antagonistic interaction. The architecture and interacting patterns between commensal and predator guilds were similar, indicating that their shared dependence on wood-dependent sub-networks determines analogous network properties. In any case, weakly connected and highly modular antagonistic and mutualistic networks are related with a high interaction intimacy [54], which effect on network architecture depends on the interaction type (mutualistic vs. antagonistic) [11], and as our results highlight also on other types of interactions.

By other side, the specialisation index (H2′) showed that insect-dependent sub-networks were more specialised in the distribution of their connections, what can be heavily determined by their dependence on the distribution and abundance of wood-dependent species. Variance-ratio showed the existence of competence patterns among species of the same feeding guild, so we can expect a stronger competence among ecologically related species coexisting in the same sub-module, as showed by Rezende et al. [52] for phylogenetically and ecologically related species among predators. Sirami et al. [18] suggested that saproxylic assemblages in Mediterranean forests are especially dependent on the availability of trophic resources at local habitat. Here, we also suggested that the distributional patterns structuring saproxylic communities were also influenced by the boundary of the interaction and interrelations occurring along functional modules housed in tree hollow microhabitats.

### Implications to Robustness in Saproxylic Networks

Saproxylic trophic levels were moderately robust to species extinction in both random and directed cascading extinction of tree hollows, being slightly more vulnerable to a random sequence of losses. In a random simulation, the high amounts of weak and heterogeneously connected insect nodes determined lower network robustness, being more sensitive to disappear with the removal of tree hollow nodes. Otherwise a directed removal of nodes gradually affected the dense distribution of these weak nodes. The high proportion of non-hubs connecting the most of nodes among sub-modules and sub-networks seemed to be conditioning relative good robustness to species extinctions, highlighting the importance of ‘effective communication’ [55] between insect species and tree hollows in the network of interactions. Stability and species coexistence of trophic networks is enhanced in modular and weakly connected architectures [4] retaining the impacts of a perturbation within a single module and minimising impacts on other modules [56]. On the contrary, food webs with a low level of modularity (densely connected species connected to each other) may confer higher robustness [57]. Accordingly, we found that sub-networks with a lower number of sub-modules, corresponding to wood-dependent sub-networks, presented higher network robustness. Finally, we observed a strong association among connectance, robustness and type of interaction. Wood-dependent feeding guilds with quite different species richness always presented higher connectance and higher robustness values than insect-dependent sub-networks of predators and commensals, for instance Dunne et al. [58] concluded that food-web robustness does not relate to species richness, but increases significantly with greater connectance. Therefore, robustness in saproxylic sub-networks seems to be conditioned by the presence of effective nodes, weak connections, a suitable number of sub-modules and the network connectance.

Usually if a portion of an ecosystem loses biodiversity as a result of some catastrophic event or severe anthropogenic modification, it will eventually regain species through linkage with adjacent ecosystems [59]. Our results highlight that saproxylic biodiversity is more dependent and specialised in trees with large holes, as in Ranius and Jansson [60], Micó et al. [29] and Gouix [53], where a higher richness and abundance of trophic resources, microhabitats or host/preys are available. These results are only focussed on the robustness according to the analysed network of interactions, and do not consider other critical characteristics characteristic to this Mediterranean forests, as isolation and low area of mature forests, or the limited proportions of tree hollows in them. Impoverishment linked to traditional habitat management based on removing old trees, dead or fallen wood, abruptly limits the microhabitat variability, and leads to habitat lost and isolation [61], affecting tree hollow-insect species interaction. Microhabitat impoverishment could also lead to an ecological disruption because of their important ecological role in forest ecosystems, i.e. fragmentation and nutrient recycling of wood decay [62] and performing in the maintenance of the trophic chains [31].

Our results emphasise the importance of the study of interrelations in understanding the distributional and interacting patterns modelling saproxylic communities in tree hollow microhabitats in Mediterranean forests. Conservation of one of the most complex and diverse terrestrial communities, such as saproxylic assemblages, needs a much better knowledge of species, processes and interactions.

## Supporting Information

Table S1 **Species list, abundance and labels.** Species list and abundance only considering saproxylic species. Saproxylic trophic guild of each species and their labels: xylophagous (C), saprophagous (A), xylomycetophagous (B), predator (E), and commensal (D).
(DOCX)

Table S2 **Node composition.** Node composition for the five main sub-modules present at the complete network, with node names and their respective labels in the diagrams. k: number of links of a node; Z-score: within-module degree of a node; P-score: Participation coefficient (between-module degree); role: ecological region.
(DOCX)
