# Correction: Breaking down Complex Saproxylic Communities: Understanding Sub-Networks Structure and Implications to Network Robustness

**DOI:** 10.1371/annotation/90da0915-2d86-4e3d-a678-b44052f80a17

**Published:** 2013-10-23

**Authors:** Javier Quinto, Ma. Ángeles Marcos-García, Cecilia Díaz-Castelazo, Víctor Rico-Gray, Hervé Brustel, Eduardo Galante, Estefanía Micó

The explanation of the algorithm used in the research from reference 49 is not expressed clearly. The interpretation of the results and discussion are the same. Here is a short list of changes to help clarify the manuscript:

- Page 3, 2nd column, 3rd paragraph, lines 6-7: Replacement of ‘of highly’ with ‘from the least to the most’

- Page 4, Table 1, Legend: line 5: Replacement of ‘most’ with ‘least’

- Page 6, 1st column, 2nd paragraph, line 8: Replacement of ‘most’ with ‘least’ 

