# Supplementary material for: Breaking down Complex Saproxylic Communities: Understanding Sub-Networks Structure and Implications to Network Robustness
Source: PLoS One. 2012 Sep 28;7(9):e45062. doi: 10.1371/journal.pone.0045062 (PMC3460928; doi:10.1371/journal.pone.0045062)
Supplement: Table S1 — Species list, abundance and labels. Species list and abundance only considering saproxylic species. Saproxylic trophic guild of each species and their labels: xylophagous (C), saprophagous (A), xylomycetophagous (B), predator (E), and commensal (D). (DOCX) [file pone.0045062.s001.docx]

**Table S1. Species list, abundance and labels.**

|  | **Abundance** | **Trophic guild label** |
| --- | --- | --- |
| **DIPTERA** |  |  |
| **Syrphidae** |  |  |
| *Brachyopa insensilis* Collin, 1939 | 10 | A1 |
| *Brachypalpoides lentus* (Meigen, 1822) | 2 | A2 |
| *Brachypalpus valgus* (Panzer, 1798) | 4 | A3 |
| *Callicera aurata* (Rossi, 1790) | 2 | A4 |
| *Callicera spinolae* Rondani, 1844 | 29 | A5 |
| *Ceriana vespiformis* (Latreille, 1804) | 9 | A6 |
| *Criorhina floccosa* (Meigen, 1822) | 8 | A7 |
| *Criorhina pachymera* Egger**,** 1858 | 4 | A8 |
| *Ferdinandea aurea* Rondani, 1844 | 24 | A9 |
| *Ferdinandea cuprea* (Scopoli, 1763) | 3 | A10 |
| *Ferdinandea fumipennis* Kassebeer, 1999 | 1 | A11 |
| *Ferdinandea ruficornis* (Fabricius, 1775) | 6 | A12 |
| *Mallota cimbiciformis* (Fallen, 1817) | 33 | A13 |
| *Mallota dusmeti* Andréu, 1926 | 97 | A14 |
| *Mallota fuciformis* (Fabricius, 1794) | 3 | A15 |
| *Milesia cabroniformis* (Fabricius, 1795) | 1 | A16 |
| *Myathropa florea* (Linnaeus, 1758) | 203 | A17 |
| *Myolepta difformis* Strobl in Czerny & Strobl, 1909 | 10 | A18 |
| *Myolepta dubia* (Fabricius, 1805) | 1 | A19 |
| *Myolepta obscura* Becher, 1882 | 4 | A20 |
| *Sphiximorpha subsessilis* (Illiger in Rossi, 1807) | 1 | A21 |
| *Spilomyia digitata* (Rondani, 1865) | 6 | A22 |
| **COLEOPTERA** |  |  |
| **Aderidae** |  |  |
| *Aderus populneus* (Panzer, 1796) | 38 | C1 |
| *Cnopus minor* (Baudi, 1877) | 1 | C2 |
| *Otolelus neglectus* (Jacquelin du Duval, 1863) | 48 | C3 |
| **Anobiidae** |  |  |
| *Dorcatoma vaulogeri agenjoi* Español, 1978 | 17 | B1 |
| *Oligomerus brunneus* (Olivier, 1790) | 28 | C4 |
| *Rhamna semen* Peyerimhoff de Fontenelle, 1913 | 27 | B2 |
| **Biphylidae** |  |  |
| *Diplocoelus fagi* Guérin-Ménéville, 1838 | 33 | B3 |
| **Buprestidae** |  |  |
| *Acmaeodera degener 14-punctata* (Scopoli, 1763) | 2 | C5 |
| *Eurythyrea quercus* (Herbst, 1780) | 1 | C6 |
| **Cerambycidae** |  |  |
| *Alocerus moesiacus* (Frivaldsky, 1838) | 15 | C7 |
| *Cerambyx welensii* (Küster, 1846) | 3 | C8 |
| *Prinobius myardi* Mulsant, 1851 | 12 | C9 |
| *Stictoleptura trisignata* (Fairmaire, 1852) | 38 | C10 |
| *Trichoferus fasciculatus* (Faldermann, 1837) | 1 | C11 |
| **Cerylonidae** |  |  |
| *Cerylon histeroides* Fabricius, 1792 | 1 | B4 |
| **Cetoniidae** |  |  |
| *Cetonia aurataeformis* Curti, 1913 | 245 | A23 |
| *Potosia cuprea* (Fabricius, 1775) | 34 | A24 |
| *Potosia opaca* (Fabricius, 1787) | 14 | A25 |
| **Ciidae** |  |  |
| *Cis striatulus* Mellie 1848 | 1 | B5 |
| *Cis villosulus* Marsham, 1802 | 1 | B6 |
| **Clambidae** |  |  |
| *Calyptomerus* sp | 1 | B7 |
| *Clambus* sp | 4 | B8 |
| **Cleridae** |  |  |
| *Opilo domesticus* (Sturm, 1837) | 7 | E1 |
| **Cryptophagidae** |  |  |
| *Atomaria (Anchicera) pusilla* (Paykull, 1798), | 2 | B9 |
| *Cryptophagus cylindrus* Kiesenwetter, 1858 | 4 | A26 |
| *Cryptophagus dentatus* (Herbst, 1793) | 23 | A27 |
| *Cryptophagus distinguendus* Sturm, 1845 | 2 | A28 |
| *Cryptophagus fallax* Balfour-Browne 1953 | 1 | A29 |
| *Cryptophagus micaceus* Rey, 1889 | 142 | E2 |
| *Cryptophagus punctipennis* Brisout de Barneville, 1863 | 54 | C12 |
| *Cryptophagus reflexus* Rey, 1889 | 268 | C13 |
| *Cryptophagus saginatus* Sturm, 1845 | 36 | B10 |
| *Cryptophagus scanicus* (Linnaeus, 1758) | 119 | B11 |
| **Curculionidae** |  |  |
| *Camptorhinus simplex* Seidlitz, 1867 | 1 | A30 |
| *Camptorhinus statua* (Rossi, 1790) | 82 | A31 |
| *Gasterocercus hispanicus* Alonso-Zarazaga, Jover y Micó, 2009 | 1 | B12 |
| **Curculionidae (Scolytiinae)** |  |  |
| *Xyleborinus saxesenii* (Ratzeburg, 1837) | 37 | B13 |
| *Xyleborus dryographus* (Ratzeburg, 1837) | 9 | B14 |
| *Xyleborus monographus* (Fabricius, 1792) | 321 | B15 |
| **Dasytidae** |  |  |
| *Aplocnemus brevis* (Rosenhauer, 1856). | 1 | E3 |
| *Aplocnemus consobrinus* (Rosenhauer, 1856) | 1 | E4 |
| *Aplocnemus limbipennis* Kiesenwetter, 1865 | 1 | E5 |
| *Mauroania* bourgeoisi (Pic, 1894) | 3 | E6 |
| **Dermestidae** |  |  |
| *Anthrenus (Anthrenus) angustefasciatus* Ganglbauer, 1904 | 3 | D1 |
| *Anthrenus (Anthrenus) festivus* Erichson, 1846 | 8 | D2 |
| *Anthrenus (Florilinus) minutus* Erichson, 1846 | 40 | D3 |
| *Anthrenus (Florilinus) verbasci* (Linnaeus, 1767) | 5 | D4 |
| *Attagenus incognitus* Hava, 2003 | 5 | D5 |
| *Attagenus schaefferi* (Herbst, 1792) | 1 | D6 |
| *Attagenus trifasciatus*  (Fabricius, 1787) | 17 | D7 |
| *Dermestes (Dermestes) bicolor* Fabricius, 1781 | 1 | D8 |
| *Dermestes (Dermestinus) erichsonii* Ganglbauer, 1904 | 1 | D9 |
| *Dermestes (Dermestinus) frischii Kugelann, 1792* | 1 | D10 |
| *Dermestes (Dermestes) hispanicus* Kalik, 1952 | 4 | D11 |
| *Dermestes (Dermestinus) undulatus* Brahm, 1790 | 22 | D12 |
| *Orphilus niger*  (Rossi, 1790) | 2 | D13 |
| **Dynastidae** |  |  |
| *Oryctes nasicornis* (Linnaeus, 1758) | 7 | A32 |
| **Elateridae** |  |  |
| *Ampedus aurilegulus* (Schaufuss, 1862) | 35 | E7 |
| *Ectamenogonus montandoni* Buysson, 1888 | 7 | E8 |
| *Elater ferrugineus* Linnaeus, 1758 | 33 | E9 |
| *Elathous platiai* Zapata & Sánchez-Ruiz 2007 | 1 | E10 |
| *Ischnodes sanguinicollis* (Panzer, 1793) | 63 | E11 |
| *Lacon punctatus* (Herbst, 1779) | 11 | E12 |
| Limoniscus violaceus (Müller, 1821) | 2 | A33 |
| Megapenthes lugens (Redtenbacher, 1842) | 20 | E13 |
| *Procraerus tibilais* (Boisduval & Lacordaire, 1835) | 12 | E14 |
| **Endomychidae** |  |  |
| *Mycetaea hirta* (Marsham, 1802) | 7 | B16 |
| *Symbiotes gibberosus* (Lucas, 1849) | 6 | B17 |
| **Eucinetidae** |  |  |
| *Nycteus meridionalis* Laporte de Castelnau, 1836 | 1 | B18 |
| **Helodidae (Scirtidae)** |  |  |
| *Prionocyphon serricornis* (Müller, 1821) | 203 | D14 |
| **Laemophloeidae** |  |  |
| *Cryptolestes ferrugineus* (Stephens, 1831) | 7 | B19 |
| *Laemophloeus nigricollis* Lucas, 1849 | 2 | B20 |
| Placonotus testaceus (Fabricius, 1787) | 2 | B21 |
| **Latridiidae** |  |  |
| *Corticaria obscura* Brisout, 1863 | 2 | D15 |
| *Dianerella ruficollis* (Marsham, 1802) | 1 | B22 |
| *Enicmus brevicornis* (Mannerheim, 1844) | 4 | B23 |
| *Enicmus rugosus* (Herbst, 1793) | 4 | B24 |
| *Lathridius assimilis* (Mannerheim, 1844) | 25 | B25 |
| **Leiodidae** |  |  |
| *Agathidium (Neoceble) nigriceps* Brisout, 1872 | 2 | B26 |
| **Lucanidae** |  |  |
| *Dorcus parallelepipedus* (Linnaeus, 1785) | 43 | C14 |
| **Malachiidae** |  |  |
| *Anthocomus fenestratus* Linder, 1864 | 16 | E15 |
| *Axinotarsus marginalis* (Laporte de Castelnau, 1840) | 6 | E16 |
| *Hypebaeus albifrons* (Fabricius, 1775) | 3 | E17 |
| *Troglops furcatus* Abeille de Perrin, 1885 | 52 | E18 |
| **Melandryidae** |  |  |
| *Orchesia micans* (Panzer, 1794) | 8 | B27 |
| **Melyridae** |  |  |
| Falsomelyris granulata (Fabricius, 1792) | 2 | E19 |
| **Mycetophagidae** |  |  |
| *Litargus balteatus* Le Conte, 1856 | 1 | B28 |
| *Litargus connexus* (Geoffroy, 1785) | 11 | B29 |
| *Mycetophagus quadriguttatus* Müller, 1821 | 73 | B30 |
| **Nitidulidae** |  |  |
| *Amphotis marginata* (Fabricius, 1781) | 2 | D16 |
| *Carpophilus* sp. | 1 | D17 |
| *Epuraea fuscicollis* (Stephens, 1832) | 137 | D18 |
| *Epuraea ocularis* Fairmaire, 1849 | 2 | D19 |
| *Soronia oblonga* C.Brisout de Barneville, 1863 | 141 | D20 |
| **Oedemeridae** |  |  |
| *Ischnomera xanthoderes* (Mulsant, 1858) | 113 | C16 |
| **Platypodidae** |  |  |
| *Platypus cylindrus* (Fabricius, 1792) | 1 | C17 |
| **Pselaphidae** |  |  |
| *Euplectus sp* | 1 | D21 |
| *Geopsephalus* | 1 | D22 |
| tribu *Goniaceritae* | 1 | E20 |
| **Ptilidae** |  |  |
| *sp 1* | 46 | B31 |
| **Ptinidae** |  |  |
| *Dignomus irroratus* (Kiesenwetter, 1851) | 4 | A34 |
| *Ptinus (Cyphoderes) bidens* Olivier, 1790 | 13 | A35 |
| *Ptinus (Cyphoderes) hirticornis* Kiesenwetter, 1867 | 3 | A36 |
| *Ptinus (Ptinus) spitzyi* Villa & Villa, 1838 | 1 | A37 |
| *Ptinus (Ptinus) timidus*  Brisout de Barneville, 1866 | 274 | C15 |
| **Rhizophagidae** |  |  |
| *Rhizophagus unicolor* Lucas, 1846 | 1 | E21 |
| **Scraptiidae** |  |  |
| *Anaspis regimbarti* Schilsky, 1895 | 11 | C18 |
| *Pentaria defarguesi* Abeille de Perrin, 1885 | 1 | C19 |
| *Scraptia schotti* Leblanc, 2010 | 2 | C20 |
| *Scraptia testacea* Allen, 1940 | 80 | C21 |
| **Scydmaenidae** |  |  |
| Cephenium (Cephenium) sp. | 1 | D23 |
| *Palaeostigus palpalis* (Latreille, 1804) | 5 | D24 |
| *Scydmaenus (Cholerus) cornutus* Motschulsky, 1845 | 3 | D25 |
| *Stenichnus (Cyrtoscydmus) godarti* (Latreille, 1806) | 5 | D26 |
| **Silvanidae** |  |  |
| *Ahasverus advena* (Waltl, 1834) | 2 | B32 |
| *Airaphilus sp.* | 1 | B33 |
| *Oryzaephilus surinamensis* (Linnaeus, 1758). | 1 | B34 |
| *Silvanus bidentatus* Fabricius, 1792 | 2 | B35 |
| *Uleiota planata* (Linnaeus, 1761) | 7 | B36 |
| **Tenebrionidae** |  |  |
| *Corticeus fasciatus* (Fabricius, 1790) | 1 | D27 |
| *Eledonoprius armatus* (Panzer, 1799) | 7 | B37 |
| *Probaticus anthracinus* (Germar, 1813) | 37 | A38 |
| *Probaticus granulatus* (Allard, 1876) | 1 | A39 |
| *Stenohelops sublinearis* (Kraatz, 1870) | 4 | A40 |
| *Tenebrio punctipennis* Seidlitz, 1896 | 33 | D28 |
| **Tenebrionidae (Alleculinae)** |  |  |
| *Isomira hispanica* Kiesenwetter, 1870 | 11 | A41 |
| Mycetochara linearis (Illiger, 1794) | 20 | A42 |
| *Mycetochara quadrimaculata* (Latreille, 1804) | 110 | A43 |
| *Prionychus fairmairei* (Reiche, 1860) | 17 | A44 |
| *Pseudocistela ceramboides* (Linnaeus, 1761) | 43 | A45 |
| **Trogossitidae** |  |  |
| *Tenebroides marrocanus* Reitter, 1884 | 3 | E23 |
| *Temnochila caerulea* (Olivier, 1790) | 3 | E24 |
| **Zopheridae** |  |  |
| *Colobicus hirtus* (Rossi, 1790) | 2 | E25 |
| *Colydium elongatum* (Fabricius, 1787) | 6 | E26 |
| *Endophloeus marcovichianus (Piller & Mitterpacher, 1783)* | 22 | B38 |

Species list and abundance only considering saproxylic species. Saproxylic trophic guild of each species and their labels: xylophagous (C), saprophagous (A), xylomycetophagous (B), predator (E), and commensal (D).
