# Supplementary material for: Breaking down Complex Saproxylic Communities: Understanding Sub-Networks Structure and Implications to Network Robustness
Source: PLoS One. 2012 Sep 28;7(9):e45062. doi: 10.1371/journal.pone.0045062 (PMC3460928; doi:10.1371/journal.pone.0045062)
Supplement: Table S2 — Node composition. Node composition for the five main sub-modules present at the complete network, with node names and their respective labels in the diagrams. k: number of links of a node; Z-score: within-module degree of a node; P-score: Participation coefficient (between-module degree); role: ecological region. (DOCX) [file pone.0045062.s002.docx]

**Table S2. Node composition.**

| **Sub-module** | **Node name** | **Label** | **k** | ***Z-score*** | ***P-score*** | **Role** |
| --- | --- | --- | --- | --- | --- | --- |
| 1 | Fr2 | 5 | 29 | 1.284.666 | 0.813317 | R4 |
|  | Fr9b | 13 | 22 | 2.893.541 | 0.735537 | R6 |
|  | Fr19 | 17 | 20 | 0.732798 | 0.765 | R3 |
|  | Fr8 | 18 | 20 | 1.758.715 | 0.685 | R3 |
|  | Fr25 | 21 | 17 | 1.758.715 | 0.602076 | R2 |
|  | Qi17 | 36 | 13 | 0.292384 | 0.686391 | R3 |
|  | VBQp8b | 38 | 13 | 0.048853 | 0.733728 | R3 |
|  | Fr9a | 45 | 11 | 0.048853 | 0.677686 | R3 |
|  | Qi23 | 47 | 11 | 0.292384 | 0.61157 | R2 |
|  | Fr16 | 56 | 9 | 0.390826 | 0.493827 | R2 |
|  | Fr26 | 57 | 9 | -0.293119 | 0.641975 | R3 |
|  | Fr9c | 64 | 8 | 0.048853 | 0.53125 | R2 |
|  | Fr21 | 69 | 7 | 0.732798 | 0 | R1 |
|  | Qi20a | 70 | 7 | -0.536036 | 0.693878 | R3 |
|  | Fr3 | 80 | 4 | -0.635092 | 0.375 | R2 |
|  | Qi18 | 85 | 3 | -0.737034 | 0.666667 | R3 |
|  | Qi19b | 86 | 3 | -0.812176 | 0.444444 | R2 |
|  | C15 | 91 | 35 | 244.266 | 0.796735 | R3 |
|  | C16 | 92 | 35 | 2.501.504 | 0.752653 | R7 |
|  | C21 | 98 | 26 | 0.732798 | 0.810651 | R4 |
|  | A43 | 100 | 23 | 107.477 | 0.763705 | R3 |
|  | E11 | 105 | 19 | 0.732798 | 0.698061 | R3 |
|  | A42 | 121 | 12 | 0.732798 | 0.611111 | R2 |
|  | A25 | 126 | 10 | 0.390826 | 0.58 | R2 |
|  | D3 | 132 | 9 | 0.390826 | 0.518519 | R2 |
|  | D7 | 133 | 9 | -0.251577 | 0.765432 | R3 |
|  | B27 | 141 | 6 | -0.631579 | 0.722222 | R3 |
|  | A41 | 143 | 6 | -0.259896 | 0.5 | R2 |
|  | C9 | 145 | 5 | -0.293119 | 0.32 | R2 |
|  | A6 | 157 | 4 | -0.635092 | 0.375 | R2 |
|  | A8 | 158 | 4 | -0.977064 | 0.625 | R3 |
|  | E6 | 168 | 3 | 0.300273 | 0 | R1 |
|  | E23 | 170 | 3 | -0.812176 | 0.444444 | R2 |
|  | D26 | 174 | 3 | -0.737034 | 0.666667 | R3 |
|  | C3 | 175 | 2 | -0.737034 | 0.5 | R2 |
|  | B32 | 181 | 2 | -0.894737 | 0.5 | R2 |
|  | C19 | 203 | 1 | -1.319.036 | 0 | R1 |
|  | C20 | 204 | 1 | -0.737034 | 0 | R1 |
|  | A16 | 218 | 1 | -0.428222 | 0 | R1 |
|  | A21 | 220 | 1 | -1.319.036 | 0 | R1 |
|  | E4 | 227 | 1 | -0.737034 | 0 | R1 |
|  | E20 | 231 | 1 | -1.319.036 | 0 | R1 |
|  | D22 | 242 | 1 | -0.428222 | 0 | R1 |
| 2 | Fr10 | 4 | 30 | 377.366 | 0.762222 | R7 |
|  | Fr23 | 6 | 28 | 107.477 | 0.795918 | R3 |
|  | Fr29 | 12 | 22 | 0.5581 | 0.785124 | R3 |
|  | VSQp7 | 14 | 22 | 1.968.036 | 0.549587 | R2 |
|  | VSQp14 | 20 | 19 | 1.498.057 | 0.542936 | R2 |
|  | VSQp4 | 23 | 17 | 0.5581 | 0.698962 | R3 |
|  | Qi2 | 26 | 15 | 0.292384 | 0.72 | R3 |
|  | VBQp15 | 27 | 15 | 0.32311 | 0.684444 | R3 |
|  | VBQp6 | 28 | 15 | 1.028.079 | 0.506667 | R2 |
|  | Qi1 | 31 | 14 | 0.754732 | 0.806122 | R4 |
|  | VBQp2 | 34 | 14 | 0.32311 | 0.693878 | R3 |
|  | Qi8 | 41 | 12 | 0.016244 | 0.680556 | R3 |
|  | VSQf16 | 50 | 11 | 0.5581 | 0.396694 | R2 |
|  | VSQp2 | 55 | 10 | -0.368421 | 0.72 | R3 |
|  | Fr17 | 74 | 6 | -0.635092 | 0.611111 | R2 |
|  | Qi12a | 78 | 5 | -0.251577 | 0.64 | R3 |
|  | C13 | 89 | 51 | 1.968.036 | 0.80892 | R4 |
|  | A23 | 90 | 39 | 1.968.036 | 0.78238 | R3 |
|  | C12 | 102 | 22 | 1.028.079 | 0.727273 | R3 |
|  | B25 | 110 | 16 | 0.088121 | 0.757813 | R3 |
|  | B10 | 115 | 14 | 0.793089 | 0.530612 | R2 |
|  | A24 | 116 | 14 | 0.754732 | 0.785714 | R3 |
|  | A45 | 117 | 14 | 0.32311 | 0.673469 | R3 |
|  | A44 | 119 | 13 | 0.32311 | 0.591716 | R2 |
|  | E7 | 139 | 7 | -0.381858 | 0.612245 | R2 |
|  | D24 | 152 | 5 | -0.851837 | 0.72 | R3 |
|  | B23 | 154 | 4 | -0.851837 | 0.625 | R3 |
|  | A34 | 160 | 4 | -0.851837 | 0.625 | R3 |
|  | A40 | 167 | 3 | -0.894737 | 0.666667 | R3 |
|  | D4 | 172 | 3 | -0.251577 | 0.444444 | R2 |
|  | C5 | 176 | 2 | -0.812176 | 0 | R1 |
|  | B20 | 178 | 2 | -0.754732 | 0.5 | R2 |
|  | B21 | 179 | 2 | -0.894737 | 0.5 | R2 |
|  | A2 | 183 | 2 | -0.851837 | 0 | R1 |
|  | A7 | 185 | 2 | -0.851837 | 0 | R1 |
|  | A9 | 186 | 2 | -0.851837 | 0 | R1 |
|  | A28 | 189 | 2 | -1.319.036 | 0.5 | R2 |
|  | E24 | 192 | 2 | -0.251577 | 0 | R1 |
|  | C2 | 198 | 1 | -0.754732 | 0 | R1 |
|  | C6 | 199 | 1 | -1.086.826 | 0 | R1 |
|  | C11 | 201 | 1 | -1.086.826 | 0 | R1 |
|  | C17 | 202 | 1 | -1.088.316 | 0 | R1 |
|  | B12 | 208 | 1 | -1.086.826 | 0 | R1 |
|  | B34 | 214 | 1 | -0.754732 | 0 | R1 |
|  | A32 | 223 | 1 | -1.086.826 | 0 | R1 |
|  | A37 | 224 | 1 | -1.086.826 | 0 | R1 |
|  | E10 | 229 | 1 | -1.088.316 | 0 | R1 |
|  | E19 | 230 | 1 | -0.754732 | 0 | R1 |
|  | E21 | 232 | 1 | -1.086.826 | 0 | R1 |
|  | D11 | 237 | 1 | -1.088.316 | 0 | R1 |
|  | D13 | 238 | 1 | -0.894737 | 0 | R1 |
|  | D21 | 241 | 1 | -0.754732 | 0 | R1 |
| 3 | VSQf12 | 8 | 27 | 1.263.068 | 0.72428 | R3 |
|  | Fr12 | 29 | 14 | 0.818927 | 0.806122 | R4 |
|  | Qi15 | 32 | 14 | 1.120.804 | 0.530612 | R2 |
|  | Fr24 | 39 | 12 | 0.251577 | 0.805556 | R4 |
|  | Qi4a | 40 | 12 | 0.016244 | 0.736111 | R3 |
|  | Qi19a | 46 | 11 | 0.292384 | 0.628099 | R3 |
|  | Qi4b | 48 | 11 | 0.568524 | 0.545455 | R2 |
|  | Fr6 | 51 | 10 | -0.293119 | 0.78 | R3 |
|  | Qi25 | 52 | 10 | 0.568524 | 0.46 | R2 |
|  | Qi3 | 53 | 10 | -0.259896 | 0.74 | R3 |
|  | Qi13 | 58 | 9 | 0.292384 | 0.518519 | R2 |
|  | Qi5a | 59 | 9 | 0.292384 | 0.493827 | R2 |
|  | Fr28 | 61 | 8 | 0.292384 | 0.375 | R2 |
|  | Fr5 | 62 | 8 | 0.048853 | 0.5625 | R2 |
|  | Fr7 | 63 | 8 | -0.635092 | 0.75 | R3 |
|  | Fr11 | 67 | 7 | -0.616847 | 0.693878 | R3 |
|  | Fr20 | 68 | 7 | -0.635092 | 0.693878 | R3 |
|  | Qi24 | 71 | 7 | 0.292384 | 0.244898 | R2 |
|  | Qi26 | 72 | 7 | 0.016244 | 0.44898 | R2 |
|  | Qi7 | 73 | 7 | -0.259896 | 0.571429 | R2 |
|  | Fr27 | 75 | 6 | 0.048853 | 0.277778 | R2 |
|  | Qi22 | 76 | 6 | 0.016244 | 0.277778 | R2 |
|  | Qi11 | 77 | 5 | -0.536036 | 0.56 | R2 |
|  | Qi14 | 79 | 5 | -0.536036 | 0.48 | R2 |
|  | Qi5b | 81 | 4 | -0.085644 | 0.5 | R2 |
|  | Qi6 | 82 | 4 | -0.251577 | 0.625 | R3 |
|  | Qi21 | 87 | 3 | -0.812176 | 0.444444 | R2 |
|  | B15 | 88 | 61 | 4.710.623 | 0.779898 | R7 |
|  | D14 | 95 | 28 | 0.844664 | 0.80102 | R4 |
|  | B3 | 96 | 27 | 2.100.687 | 0.748971 | R3 |
|  | A17 | 99 | 24 | 1.416.743 | 0.756944 | R3 |
|  | B13 | 103 | 22 | 0.818927 | 0.805785 | R4 |
|  | B2 | 109 | 16 | 0.844664 | 0.671875 | R3 |
|  | C10 | 114 | 14 | 0.568524 | 0.673469 | R3 |
|  | C7 | 124 | 11 | 1.120.804 | 0.31405 | R2 |
|  | A5 | 125 | 10 | 0.568524 | 0.48 | R2 |
|  | A14 | 130 | 9 | 0.568524 | 0.345679 | R2 |
|  | A18 | 137 | 7 | -0.251577 | 0.77551 | R3 |
|  | E1 | 138 | 7 | 0.016244 | 0.44898 | R2 |
|  | A13 | 142 | 6 | -0.259896 | 0.444444 | R2 |
|  | D2 | 151 | 5 | -0.851837 | 0.72 | R3 |
|  | B8 | 153 | 4 | -0.259896 | 0 | R1 |
|  | B6 | 163 | 1 | -0.737034 | 0 | R1 |
|  | A36 | 166 | 3 | -0.737034 | 0.666667 | R3 |
|  | E16 | 169 | 3 | -0.812176 | 0.444444 | R2 |
|  | B5 | 206 | 1 | -1.088.316 | 0 | R1 |
|  | B7 | 207 | 1 | -1.319.036 | 0 | R1 |
|  | B16 | 209 | 1 | -0.085644 | 0.444444 | R2 |
|  | B22 | 211 | 1 | -1.088.316 | 0 | R1 |
|  | B35 | 215 | 1 | -1.319.036 | 0 | R1 |
|  | A39 | 225 | 1 | -1.088.316 | 0 | R1 |
|  | E3 | 226 | 1 | -1.088.316 | 0 | R1 |
|  | D9 | 235 | 1 | -1.086.826 | 0 | R1 |
| 4 | VBQp3 | 2 | 43 | 3.578.947 | 0.756084 | R7 |
|  | VSQf15 | 7 | 28 | 1.210.526 | 0.80102 | R4 |
|  | VBQp14 | 10 | 25 | 0.684211 | 0.784 | R3 |
|  | VBQp1 | 15 | 21 | 0.5581 | 0.770975 | R3 |
|  | VSQf13 | 16 | 20 | 2.263.158 | 0.55 | R2 |
|  | VSQp10 | 19 | 19 | 0.390826 | 0.803324 | R4 |
|  | VSQf9 | 22 | 17 | 0.016244 | 0.795848 | R3 |
|  | VSQp1 | 24 | 16 | 0.421053 | 0.742188 | R3 |
|  | Fr22 | 30 | 14 | 0.754732 | 0.826531 | R4 |
|  | VSQf6 | 35 | 14 | 0.599511 | 0.806122 | R4 |
|  | VBQp5 | 37 | 13 | -0.105263 | 0.745562 | R3 |
|  | VBQp17 | 42 | 12 | 0.684211 | 0.611111 | R2 |
|  | VBQp7 | 43 | 12 | 133.758 | 0.680556 | R3 |
|  | VBQp16 | 49 | 11 | 0.947368 | 0.446281 | R2 |
|  | VSQf5 | 54 | 10 | 133.758 | 0.66 | R3 |
|  | Qi20b | 65 | 8 | -0.536036 | 0.71875 | R3 |
|  | VBQp12 | 66 | 8 | -0.368421 | 0.71875 | R3 |
|  | Qi12b | 83 | 3 | -0.631579 | 0.444444 | R2 |
|  | A31 | 93 | 29 | 1.210.526 | 0.763377 | R3 |
|  | B11 | 94 | 28 | 0.421053 | 0.811224 | R4 |
|  | D20 | 97 | 27 | 1.210.526 | 0.776406 | R3 |
|  | E9 | 107 | 17 | 0.048853 | 0.816609 | R4 |
|  | D18 | 108 | 17 | -0.21838 | 0.795848 | R3 |
|  | B30 | 111 | 16 | 0.088121 | 0.75 | R3 |
|  | A27 | 112 | 16 | 0.300273 | 0.84375 | R4 |
|  | A38 | 113 | 16 | 1.473.684 | 0.570313 | R2 |
|  | C4 | 120 | 12 | 0.157895 | 0.736111 | R3 |
|  | E15 | 122 | 12 | 0.421053 | 0.680556 | R3 |
|  | A35 | 127 | 10 | 0.256933 | 0.78 | R3 |
|  | E2 | 128 | 10 | 0.157895 | 0.68 | R3 |
|  | B38 | 129 | 9 | 0.157895 | 0.567901 | R2 |
|  | E12 | 131 | 9 | 0.251577 | 0.765432 | R3 |
|  | A1 | 135 | 8 | -0.105263 | 0.625 | R3 |
|  | B1 | 146 | 5 | -0.631579 | 0.72 | R3 |
|  | B19 | 148 | 5 | -0.631579 | 0.64 | R3 |
|  | B29 | 149 | 5 | -0.21838 | 0.72 | R3 |
|  | B36 | 150 | 5 | -0.251577 | 0.64 | R3 |
|  | B24 | 155 | 4 | -0.368421 | 0.375 | R2 |
|  | A3 | 156 | 4 | -0.977064 | 0.625 | R3 |
|  | A22 | 159 | 4 | -0.251577 | 0.625 | R3 |
|  | D5 | 162 | 4 | -0.259896 | 0 | R1 |
|  | B37 | 182 | 2 | -0.631579 | 0 | R1 |
|  | A4 | 184 | 2 | -0.631579 | 0 | R1 |
|  | A20 | 187 | 2 | -0.737034 | 0.5 | R2 |
|  | A26 | 188 | 2 | -0.737034 | 0.5 | R2 |
|  | D15 | 196 | 2 | -0.428222 | 0.5 | R2 |
|  | B4 | 205 | 1 | -0.894737 | 0 | R1 |
|  | B33 | 213 | 1 | -1.088.316 | 0 | R1 |
|  | A12 | 217 | 1 | -0.894737 | 0 | R1 |
|  | A29 | 221 | 1 | -0.894737 | 0 | R1 |
|  | A30 | 222 | 1 | -0.894737 | 0 | R1 |
|  | D17 | 239 | 1 | -0.894737 | 0 | R1 |
|  | D19 | 240 | 1 | -0.428222 | 0 | R1 |
|  | D23 | 243 | 1 | -0.754732 | 0 | R1 |
|  | D27 | 244 | 1 | -0.894737 | 0 | R1 |
| 5 | VSQp8 | 1 | 46 | 4.367.864 | 0.810019 | R7 |
|  | VBQp10 | 3 | 37 | 2.438.015 | 0.749452 | R3 |
|  | Qi9 | 9 | 25 | 0.793089 | 0.768 | R3 |
|  | VSQf3 | 11 | 23 | 0.947368 | 0.73724 | R3 |
|  | Fr18 | 25 | 15 | 0.793089 | 0.577778 | R2 |
|  | VBQp13 | 33 | 14 | -0.259896 | 0.765306 | R3 |
|  | VBQp8a | 44 | 12 | -0.146868 | 0.722222 | R3 |
|  | Fr1 | 60 | 8 | -0.635092 | 0.65625 | R3 |
|  | Qi16 | 84 | 3 | -0.812176 | 0.444444 | R2 |
|  | C1 | 101 | 22 | 1.263.068 | 0.702479 | R3 |
|  | E18 | 104 | 20 | 1.120.804 | 0.715 | R3 |
|  | B31 | 106 | 17 | -0.085644 | 0.795848 | R3 |
|  | C14 | 118 | 13 | 0.088121 | 0.674556 | R3 |
|  | D28 | 123 | 12 | -0.146868 | 0.722222 | R3 |
|  | B14 | 134 | 8 | -0.368421 | 0.71875 | R3 |
|  | E14 | 136 | 8 | -0.293119 | 0.65625 | R3 |
|  | C18 | 140 | 6 | -0.616847 | 0.666667 | R3 |
|  | E13 | 144 | 6 | -0.146868 | 0.277778 | R2 |
|  | B17 | 147 | 5 | 0.256933 | 0.56 | R2 |
|  | E8 | 161 | 4 | -0.977064 | 0.625 | R3 |
|  | A10 | 164 | 3 | -0.428222 | 0.666667 | R3 |
|  | A15 | 165 | 3 | -0.631579 | 0.444444 | R2 |
|  | E26 | 171 | 3 | -0.634787 | 0.666667 | R3 |
|  | D25 | 173 | 3 | -0.851837 | 0.444444 | R2 |
|  | B9 | 177 | 2 | -0.851837 | 0 | R1 |
|  | B26 | 180 | 2 | -0.428222 | 0.5 | R2 |
|  | A33 | 190 | 2 | -0.737034 | 0.5 | R2 |
|  | E17 | 191 | 2 | -0.428222 | 0.5 | R2 |
|  | E25 | 193 | 2 | -0.428222 | 0.5 | R2 |
|  | D1 | 194 | 2 | -1.319.036 | 0.5 | R2 |
|  | D12 | 195 | 2 | -0.428222 | 0.5 | R2 |
|  | D16 | 197 | 2 | -0.428222 | 0.5 | R2 |
|  | C8 | 200 | 1 | -0.428222 | 0 | R1 |
|  | B18 | 210 | 1 | -1.086.826 | 0 | R1 |
|  | B28 | 212 | 1 | -0.428222 | 0 | R1 |
|  | A11 | 216 | 1 | -0.428222 | 0 | R1 |
|  | A19 | 219 | 1 | -0.894737 | 0 | R1 |
|  | E5 | 228 | 1 | -1.086.826 | 0 | R1 |
|  | D6 | 233 | 1 | -0.428222 | 0 | R1 |
|  | D8 | 234 | 1 | -0.428222 | 0 | R1 |
|  | D10 | 236 | 1 | -1.086.826 | 0 | R1 |
|  |  |  |  |  |  |  |
|  |  |  |  |  |  |  |
|  |  |  |  |  |  |  |

Node composition for the five main sub-modules present at the complete network, with node names and their respective labels in the diagrams. k: number of links of a node; Z-score: within-module degree of a node; P-score: Participation coefficient (between-module degree); role: ecological region.
